# Extracorporeal Life Support in Severe Accidental Hypothermia: Mechanisms, Challenges and Clinical Horizons

**DOI:** 10.3390/jcm15083119

**Published:** 2026-04-19

**Authors:** Debora Emanuela Torre, Carmelo Pirri

**Affiliations:** 1 Department of Cardiac Anesthesia and Intensive Care Unit, Cardiac Surgery, Ospedale dell’Angelo, Mestre, 30174 Venice, Italy; 2 Department of Neurosciences, Institute of Human Anatomy, University of Padova, 35121 Padua, Italy; carmelo.pirri@unipd.it

**Keywords:** hypothermic cardiac arrest, accidental hypothermia, extracorporeal life support, veno-arterial ECMO, extracorporeal rewarming

## Abstract

Severe accidental hypothermia represents a unique and potentially reversible cause of cardiac arrest in which prolonged resuscitation may still result in favorable neurological recovery. Unlike normothermic cardiac arrest, hypothermic cardiac arrest (HCA) is characterized by profound metabolic suppression and temperature-mediated myocardial instability, requiring a fundamentally different therapeutic paradigm. Veno-arterial extracorporeal membrane oxygenation (V-A ECMO) provides not only circulatory support but also controlled reperfusion and rewarming, positioning it as the cornerstone of modern management. Recent international guidelines have clarified indications for extracorporeal life support (ECLS) in HCA and have contributed to improved standardization of care. Building upon these recommendations, this narrative review focuses on physiological principles underlying extracorporeal rewarming and their implications for bedside management. We examine mechanisms of ischemia–reperfusion injury, rewarming-associated hemodynamic instability and myocardial stunning, discuss dynamic risk assessment beyond statistical thresholds such as the HOPE score and summarize practical considerations regarding cannulation strategies, differential hypoxia, left ventricular unloading and neurologic evaluation. By integrating current evidence with pathophysiological insight and organizational considerations, this review proposes a clinically oriented framework to support decision-making in hypothermic cardiac arrest and to optimize meaningful neurological recovery.

## 1. Introduction

Severe accidental hypothermia remains a rare but clinically distinctive cause of cardiac arrest, characterized by a paradoxical coexistence of extreme physiological derangement and potential reversibility. In contrast to normothermic cardiac arrest, where prolonged no-flow and low-flow states are strongly associated with irreversible neurological injury, hypothermic cardiac arrest (HCA) occurs in the setting of profound metabolic suppression [1]. Reduced cellular oxygen demand, slowed enzymatic activity and temperature-dependent electrophysiological instability create a pathophysiological scenario in which extended resuscitative efforts may still result in meaningful neurological recovery [2]. This unique biological context challenges conventional paradigms of cardiac arrest management. Over the past decades, veno-arterial extracorporeal membrane oxygenation (V-A ECMO) has emerged as the most effective modality for rewarming patients with HCA, providing both circulatory support and extracorporeal oxygenation while allowing controlled temperature restoration [3]. Observational data and registry analyses have demonstrated survival rates and neurological outcomes that surpass those typically observed in normothermic extracorporeal cardiopulmonary resuscitation (ECPR) [4,5]. Consequently, recent international recommendations have standardized indications for extracorporeal life support (ECLS) in severe hypothermia, emphasizing risk stratification tools such as the hypothermia outcome prediction after ECLS (HOPE) score and discouraging the application of conventional ECPR exclusion criteria to hypothermic patients [1,3,6]. However, while indications for extracorporeal rewarming have become increasingly defined, important questions remain regarding the physiological optimization of V-A ECMO in this specific setting. Hypothermic cardiac arrest is not merely a thermal emergency but a complex reperfusion scenario in which ischemia–reperfusion injury, myocardial stunning, vasoplegia, microcirculatory dysfunction and rewarming-associated hemodynamic instability interact dynamically [7]. The optimal management of rewarming kinetics, perfusion targets, oxygenation strategies, left ventricular unloading and neurologic monitoring is incompletely addressed in current consensus documents. Moreover, the integration of prehospital triage, regional ECMO networks and post-rewarming critical care requires a coordinated, system-level approach. Unlike prior narrative reviews and guideline-based documents, this work aims to move beyond descriptive synthesis of evidence by integrating pathophysiological mechanisms, clinical data and system-level organization into a unified conceptual framework. In particular, we conceptualize extracorporeal rewarming not merely as a technical intervention, but as a time-dependent, system-driven process in which outcomes emerge from the dynamic interaction between physiology, logistics and decision-making under uncertainty. This perspective also allows us to address organizational and ethical dimensions that remain underrepresented in the existing literature. In this context, the present narrative review aims to integrate current evidence with pathophysiological insight to provide a clinically oriented framework for the management of hypothermic cardiac arrest supported by V-A ECMO. Rather than reiterating guideline recommendations, we focus on the mechanisms that underlie extracorporeal resuscitation, the practical implications for bedside management and the organizational strategies necessary to translate evidence into standardized yet adaptable rescue pathways. By reframing extracorporeal rewarming as a process of controlled reperfusion within a metabolically suppressed state, we seek to support clinicians in maximizing survival while preserving neurological integrity.

## 2. Materials and Methods

This narrative review integrates current evidence regarding the use of V-A ECMO in HCA. A structured literature search was conducted in PubMed/MEDLINE, Scopus and Embase for studies published between January 1990 and February 2026, using combinations of the following terms: “hypothermic cardiac arrest”, “accidental hypothermia”, “extracorporeal life support”, “ECMO”, and “extracorporeal cardiopulmonary resuscitation”. Reference lists of selected articles and recent international recommendations were also screened to identify additional relevant publications. Observational studies, registry analyses, systematic reviews and international consensus documents were considered. Mechanistic studies relevant to hypothermia and reperfusion physiology were included when pertinent. Given the heterogeneity of available data, findings were synthesized narratively rather than through formal meta-analysis. In addition to summarizing published evidence, this review proposes a physiology-guided framework for extracorporeal resuscitation based on integration of the current literature and clinical reasoning.

## 3. Results

### 3.1. Pathophysiology of Hypothermic Cardiac Arrest

**Figure 1:**
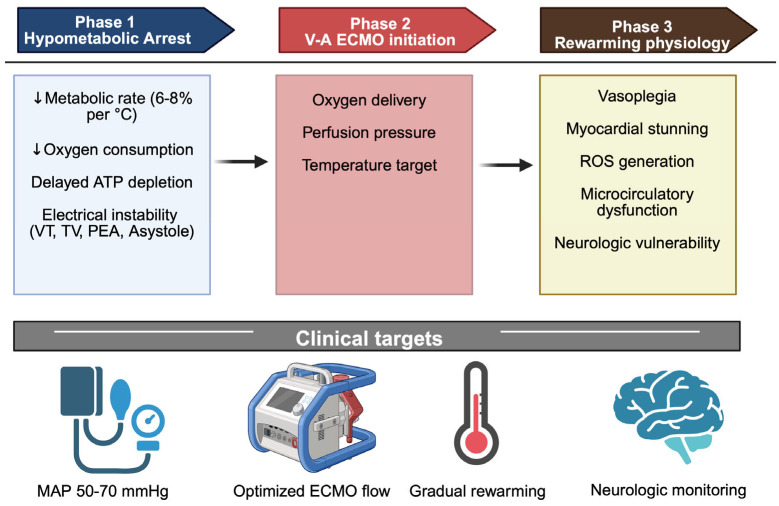
Pathophysiological model of hypothermic cardiac arrest and controlled extracorporeal reperfusion. Down arrows: increase. Created in BioRender. Pirri, C. (2026) https://BioRender.com/8by91dk.

Hypothermic cardiac arrest (HCA) represents a biologically distinct form of circulatory collapse in which temperature-dependent metabolic suppression profoundly modifies the temporal relationship between ischemia and irreversible cellular injury (Figure 1). Unlike normothermic cardiac arrest, where neuronal viability rapidly declines in proportion to oxygen deprivation, progressive hypothermia markedly reduces cerebral metabolic rate and oxygen consumption in a temperature-dependent manner, delaying adenosine triphosphate (ATP) depletion and slowing excitotoxic cascades [8]. Consequently, prolonged no-flow or low-flow states may be compatible with neurological recovery when effective reperfusion is eventually achieved [1]. At the cardiovascular level, hypothermia induces progressive electrophysiological instability. Cooling alters transmembrane ion channel kinetics, prolongs action potential duration and increases heterogeneity of myocardial repolarization, predisposing to ventricular arrhythmias. Below 30 °C, sinus bradycardia, atrial fibrillation and ventricular fibrillation become increasingly common. At temperature <28 °C, asystole may occur. Concomitantly, myocardial contractility declines due to impaired calcium handling, reduced beta-adrenergic responsiveness and altered myofilament sensitivity. The resulting decrease in cardiac output is compounded by increased systemic vascular resistance in early hypothermia, followed by vasoplegia during rewarming [7,9,10,11]. Importantly, HCA should not be conceptualized solely as a consequence of pump failure but as a systemic metabolic arrest state. Severe hypothermia affects mitochondrial oxidative phosphorylation, promotes intracellular acidosis and alters cellular redox balance. During the hypothermic phase, reduced metabolic flux may exert a protective effect; however, restoration of normothermia and oxygenated blood flow triggers a complex ischemia–reperfusion response [7]. Rewarming is associated with reactive oxygen species generation, endothelial activation, capillary leak and inflammatory mediator release. This “rewarming shock” may manifest as vasodilatory hypotension, myocardial stunning and impaired microcirculatory perfusion despite adequate microcirculatory parameters [2,12,13]. Cerebral pathophysiology in HCA reflects the interplay between metabolic suppression and reperfusion injury. Hypothermia decreases cerebral metabolic rate and may preserve neuronal viability during prolonged arrest. Nevertheless, rapid or poorly controlled rewarming can disrupt cerebral autoregulation, promote blood–brain barrier dysfunction and exacerbate oxidative stress [7,8,12,14]. Thus, neurological outcome depends not only on the duration of circulatory arrest but also on the quality and modulation of reperfusion. These mechanisms support a conceptual shift in the management of HCA: extracorporeal rewarming should be understood as a controlled reperfusion within a metabolically suppressed organism rather than simple thermal correction. V-A ECMO provides a unique platform to modulate perfusion pressure, oxygen delivery, temperature gradients and acid-base management, thereby influencing the balance between cellular recovery and reperfusion injury [1,3,15]. Understanding these pathophysiological principles is essential to optimize extracorporeal strategies and to individualize treatment beyond temperature-based thresholds alone.

### 3.2. Patient Selection and Dynamic Risk Stratification Beyond Standard Cut-Offs

**Figure 2:**
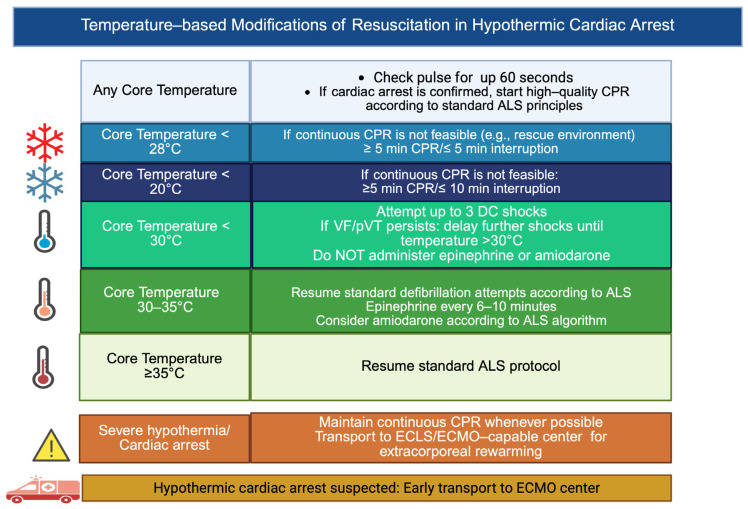
Temperature-adapted resuscitation strategy in hypothermic cardiac arrest. ALS: advanced life support; CPR: cardiopulmonary resuscitation; DC shock: direct current shock; VF: ventricular fibrillation; pVT: pulseless ventricular tachycardia; ECLS: extracorporeal life support; ECMO: extracorporeal membrane oxygenation. Created in BioRender. Pirri, C. (2026). https://BioRender.com/sc8l5a7.

Appropriate patient selection remains central to the successful use of V-A ECMO in hypothermic cardiac arrest. Recent recommendations emphasize the use of structured prognosis tools, most notably the hypothermia outcome prediction after ECLS (HOPE) score, to estimate survival probability and guide decision-making. The HOPE model integrates core temperature, serum potassium, age, sex, mechanism of hypothermia and duration of cardiopulmonary resuscitation to generate an individual survival estimate. A predicted survival probability ≥10% is commonly proposed as a pragmatic threshold to support extracorporeal rewarming [5,6]. While such models represent a major advance toward objective risk stratification, patient selection in HCA should not rely exclusively on statistical cut-offs. Hypothermic arrest is characterized by dynamic physiological transitions, and single-point laboratory or clinical values may incompletely capture the patient’s biological trajectory. For example, serum potassium, historically used as a marker of cellular lysis and non-survivability, may be influenced by sampling site, hemolysis or delayed measurement and should be interpreted in the broader clinical context [16]. Similarly, prolonged no-flow or low-flow times, asystole or advanced age, traditionally regarded as exclusion criteria in normothermic ECPR, have demonstrated limited discriminatory value in hypothermic cohorts [6]. Dynamic risk assessment should therefore integrate the following three complementary domains: mechanism and circumstances of hypothermia (e.g., avalanche burial, cold-water immersion, and urban exposure); temporal factors including witnessed status and quality of CPR; and evolving physiological parameters such as lactate trends, acid-base status, end-tidal carbon dioxide and early response to controlled reperfusion [3,5,17,18] (Table 1). Because hypothermic cardiac arrest follows pathophysiological principles distinct from normothermic arrest, resuscitation strategies must also be adapted according to core temperature and the feasibility of extracorporeal support [1] (Figure 2). This multidimensional approach acknowledges that hypothermic cardiac arrest represents a potentially reversible metabolic state rather than a uniformly progressive ischemic injury. In addition, institutional and system-level variables, including time to ECMO initiation, availability of specialized teams and transport logistics, substantially influence the outcome and must be incorporated into real-world decision-making [19]. In selected borderline cases, particularly when prognostic uncertainty is high and no clear contraindications exist, the threshold for ECLS may reasonably be individualized rather than rigidly applied. Thus, beyond standardized eligibility criteria, patient selection in HCA should be viewed as a process of dynamic risk stratification that balances biological plausibility of recovery, quality of reperfusion strategy and system capability. Such an approach may better reflect the complexity of hypothermic cardiac arrest and support ethically sound, physiology-informed decisions.

#### Limitations of Conventional Arrest Criteria

Conventional exclusion criteria derived from normothermic cardiac arrest should not be directly extrapolated to HCA. Unwitnessed arrest, prolonged no-flow or low-flow intervals, initial asystole, low end-tidal CO_2_ values, fixed dilated pupils or advanced age, traditionally associated with poor outcomes, have limited discriminatory value in the context of profound hypothermia. Therefore, the absence of witnessed collapse or extended resuscitation duration alone should not automatically preclude consideration of extracorporeal support, provided that no non-survivable cause is identified and that the overall physiological context remains compatible with reversibility [1,3,6].

### 3.3. Cannulation Strategy and Hemodynamic Targets

V-A ECMO represents the preferred modality for extracorporeal rewarming in hypothermic cardiac arrest, as it provides both systemic perfusion and oxygenation while allowing controlled temperature restoration. However, optimal outcomes depend not only on timely initiation but also on appropriate cannulation strategy and physiology-guided hemodynamic management [1,3].

#### 3.3.1. Cannulation Strategy

In emergency settings, the femoro-femoral configuration is generally the most practical and rapidly deployable approach. It can be established percutaneously under ultrasound guidance, often without interrupting cardiopulmonary resuscitation, and allows immediate initiation of extracorporeal flow [20,21]. The routine placement of a distal perfusion cannula is strongly recommended to mitigate risk of limb ischemia, particularly given the vasoconstricted and coagulopathic state typical of severe hypothermia [22]. Cannulation in hypothermic patients may be technically challenging. Peripheral vasoconstriction, increased blood viscosity and hypothermia-associated coagulopathy increase the risk of vascular injury and bleeding. Whenever feasible, percutaneous techniques should be favored over surgical cutdown to minimize tissue trauma. Brief, controlled pauses in chest compressions may be necessary during critical steps such as vessel puncture or guidewire advancement, but interruptions should otherwise be minimized [6,23,24]. Although femoro-femoral V-A ECMO is standard, clinicians must anticipate the potential development of differential hypoxia (Harlequin phenomenon or dual circulation) once native cardiac function begins to recover. In patients with impaired pulmonary gas exchange, such as those with aspiration or avalanche-related lung injury, the upper body may receive poorly oxygenated blood from the native left ventricle. Continuous monitoring of right radial arterial oxygenation saturation and arterial blood gases is therefore essential. In cases of significant differential hypoxia, conversion to a veno-arteriovenous (VAV) configuration should be considered [25,26]. Left ventricular distension during V-A ECMO in HCA should not be considered negligible and may occur depending on the degree of myocardial dysfunction and loading conditions. Although myocardial electrical stability and contractility often improve with rewarming and adequate oxygenation, leading to recovery of aortic valve opening and forward ejection, the need for unloading remains variable. Decisions regarding left ventricular decompression should therefore be individualized and guided by patient-specific hemodynamic and echocardiographic findings [6]. Left-sided distension may occur in selected cases and should be actively surveilled. Venting should be considered according to standard criteria, particularly in the presence of persistent aortic valve closure with progressive LV dilation, rising left atrial pressures, pulmonary venous congestion or refractory pulmonary edema. Bedside echocardiography is central for early recognition, enabling assessment of ventricular size, aortic valve opening and pulmonary venous congestion. When unloading is deemed necessary, the choice of strategy (medical optimization, atrial decompression or mechanical unloading) should be individualized to the patient’s hemodynamic profile and institutional expertise [21,27,28].

#### 3.3.2. Hemodynamic Targets

HCA should be conceptualized as a controlled reperfusion scenario. Therefore, hemodynamic management during V-A ECMO should aim not merely to restore circulation but to optimize oxygen delivery while limiting reperfusion injury.

- Mean Arterial Pressure (MAP)

Current recommendations suggest maintaining an MAP between 50 and 70 mmHg. In practice, targets should be individualized based on cerebral oxygenation, lactate clearance and evidence of end-organ perfusion. Excessively high perfusion pressures may increase afterload and myocardial wall stress, whereas inadequate pressure risks impaired cerebral perfusion during a vulnerable reperfusion phase [29,30,31].

- Flow targets and afterload management

Extracorporeal flow must be sufficient to maintain systemic perfusion while avoiding unnecessary increases in aortic afterload. As rewarming progresses and partial myocardial recovery occurs, careful echocardiographic assessment is essential to evaluate aortic valve opening, ventricular size and pulsatility. The need for left ventricular unloading during HCA remains variable and should be assessed on a case-by-case basis. While myocardial contractility often improves with rewarming and oxygenation, unloading strategies should be considered when hemodynamic and echocardiographic findings indicate impaired ventricular ejection or elevated filling pressures [6,28].

- Cerebral monitoring

In FF V-A ECMO right radial arterial sampling reflects cerebral oxygenation. Differential hypoxia (or dual circulation) should be actively monitored, particularly in patients with pulmonary injury. Near-infrared spectroscopy (NIRS), when available, may provide additional information on cerebral perfusion adequacy. Overall, hemodynamic management during V-A ECMO in HCA should prioritize stable systemic perfusion, avoidance of excessive afterload and continuous reassessment of native cardiac recovery [6,25,31,32].

### 3.4. Physiology-Guided Rewarming

Rewarming in HCA should not be conceptualized as a purely thermal intervention, but rather a process of controlled systemic reperfusion within a metabolically suppressed organism. V-A ECMO provides a unique platform to regulate temperature, oxygen delivery, perfusion pressure and acid-base balance simultaneously. Therefore, the objective of extracorporeal rewarming extends beyond achieving normothermia and includes mitigation of reperfusion injury and hemodynamic instability [3] (Table 2).

#### 3.4.1. Rewarming Rate and Thermal Gradients

Current recommendations allow rewarming rates up to 5 °C per hour until a core temperature of approximately 30 °C or return of spontaneous circulation. However, the biological response to rewarming remains uncertain, as available evidence is largely derived from observational data. A degree of clinical equipoise persists regarding the balance between rapid rewarming and potential risks. The biological response to rewarming is not linear and rapid temperature shifts may amplify oxidative stress, endothelial activation and microvascular dysfunction [1]. For this reason, strict control of thermal gradients within the extracorporeal circuit is essential. The temperature difference between venous inflow and arterial outlet should not exceed 4 °C to reduce the risk of hemolysis and neurological injury. In addition, the heater-set temperature should not exceed 10 °C above the venous inflow temperature and should remain below a maximum of 42 °C. Failure to adhere to these limits may result in excessive thermal stress, red blood cell damage and adverse neurological effects [6]. After reaching 30 °C, further temperature normalization should be individualized, balancing myocardial recovery, systemic vascular tone and cerebral autoregulation. Rewarming should be considered a dynamic phase in which physiological parameters, not temperature alone, guide progression.

#### 3.4.2. Hemodynamic Instability and “Rewarming Shock”

Rewarming is frequently associated with systemic vasodilation and capillary leak, leading to hypotension despite adequate extracorporeal flow. This phenomenon, often referred to as rewarming shock, reflects a combination of inflammatory activation, endothelial dysfunction and impaired vascular responsiveness. Fluid administration with warmed isotonic crystalloids and vasopressor support are commonly required. Importantly, vasoplegia may coexist with myocardial stunning, necessitating careful titration of inotropes and afterload management. Perfusion pressure should be interpreted in the context of vascular responsiveness and myocardial recovery. Excessively aggressive vasopressor use may increase left ventricular afterload, whereas insufficient perfusion pressure risks inadequate cerebral perfusion during a vulnerable reperfusion phase [7,9,14,33].

#### 3.4.3. Oxygen Delivery and Metabolic Coupling

Hypothermia markedly reduces systemic oxygen consumption (VO_2_) and early rewarming occurs in a state of relative metabolic suppression [34]. Consequently, oxygen delivery (DO_2_) should be titrated to match evolving metabolic demand rather than normalized immediately to normothermic targets [35]. Monitoring of lactate kinetics, central or mixed venous oxygen saturation and cerebral near-infrared spectroscopy (NIRS) can provide insight into tissue-level perfusion adequacy [31,36]. The transition from hypometabolic arrest to reperfused circulation represents a critical window during which excessive hyperoxia or hyperperfusion may contribute to reactive oxygen species generation. Avoidance of both hypoxia and unnecessary hyperoxia is therefore advisable, with right radial artery sampling providing a surrogate of cerebral oxygenation in FF V-A ECMO [37].

#### 3.4.4. Acid-Base Management

Alpha-stat management is generally preferred during accidental hypothermia, as it preserves cerebral autoregulation and avoids excessive cerebral blood flow. As rewarming progresses, shifts in carbon dioxide solubility and hemoglobin-oxygen affinity alter systemic and cerebral perfusion dynamics. Gradual correction of acidosis is often observed with restoration of perfusion; aggressive buffering strategies are rarely required unless severe metabolic derangements persist [6,38].

#### 3.4.5. Consideration of Discontinuation

If return of spontaneous circulation has not occurred after rewarming to 32–35 °C, consideration should be given to discontinuation of extracorporeal support. This decision should incorporate additional clinical factors, including uncontrollable hemorrhage, newly identified non-survivable causes of arrest or clear evidence of severe anoxic brain injury. Temperature alone should not be the sole determinant [6,39,40].

### 3.5. Neurologic Prognostication and Post-Rewarming Care

Neurologic outcome represents the primary determinant of meaningful survival after hypothermic cardiac arrest. Unlike normothermic cardiac arrest, however, neuroprognostication in HCA is uniquely challenging. Profound hypothermia exerts intrinsic neuroprotective effects by reducing cerebral metabolic rate, suppressing excitotoxic cascades and delaying ATP depletion. At the same time, prolonged low-flow states and subsequent reperfusion injury introduce substantial uncertainty. Consequently, premature prognostic conclusions should be strictly avoided [41,42].

#### 3.5.1. Timing of Neurologic Assessment

In HCA supported with V-A ECMO, reliable neurologic assessment should be deferred until normothermia is achieved; sedative and neuromuscular blocking agents are metabolized or discontinued; hemodynamic stability is restored. Temperature-dependent drug metabolism, altered protein binding and impaired hepatic and renal function during hypothermia may significantly prolong sedative clearance. Therefore, conventional timelines used in normothermic post-cardiac arrest care cannot be directly extrapolated to HCA. Multimodal prognostication is recommended and early clinical signs alone, such as absent motor response or brainstem reflexes, should not be considered definitive in the immediate post-rewarming phase [43,44,45].

#### 3.5.2. Multimodal Neurologic Examination

A multimodal approach integrating clinical, electrophysiological, biochemical and imaging data is advisable [41].

- Clinical examination

Serial neurologic examinations remain fundamental but must be interpreted cautiously. Pupillary reactivity, corneal reflexes and motor response should be assessed only after exclusion of confounders such as residual sedation, metabolic disturbances or severe hemodynamic instability [41,43,45,46].

- Electroencephalography (EEG)

Continuous or intermittent EEG monitoring may help detect seizures, non-convulsive status epilepticus or highly malignant patterns. Early EEG abnormalities may reflect transient metabolic suppression rather than irreversible injury; thus, trends over time are more informative than single recordings [43,47,48,49].

- Biomarkers

Neuron-specific enolase (NSE) and S100B protein may provide adjunctive information; however, their interpretation in hypothermic arrest remains complex. Rather than relying on absolute thresholds, serial trends of these biomarkers may offer more informative insights, particularly in the context of ECMO where hemolysis and altered clearance may confound serum levels. Therefore, biomarker values should be interpreted within a multimodal framework, and trends over time should be prioritized over isolated measurements. Therefore, biomarker thresholds validated in normothermic cardiac arrest should not be applied uncritically to HCA [50,51,52].

- Neuroimaging

Brain computed tomography (CT) is useful to exclude catastrophic intracranial pathology. Magnetic resonance imaging (MRI), when feasible after hemodynamic stabilization, may detect diffusion restriction suggestive of anoxic injury. Imaging findings should be integrated with clinical and electrophysiological data rather than interpreted in isolation [53].

#### 3.5.3. Post-Rewarming Intensive Care Management

After rewarming and restoration of spontaneous circulation or stable extracorporeal support, patients should receive comprehensive post-cardiac arrest care [48].

- Oxygenation and ventilation

Both hypoxia and hyperoxia should be avoided. Protective lung ventilation strategies are recommended, particularly in patients with aspiration or avalanche-related injury [43,48].

- Hemodynamic optimization

Persistent myocardial stunning, vasoplegia or combined cardiogenic-distributive shock may occur. Hemodynamic management should aim to maintain adequate cerebral perfusion while avoiding excessive afterload that could impair ventricular recovery [48,54].

- Temperature management after normothermia

Once normothermia is achieved, strict avoidance of hyperthermia is essential. Whether targeted temperature management confers additional benefit in hypothermic cardiac arrest remains uncertain; however, uncontrolled fever may exacerbate secondary brain injury [55].

- Seizure management

Electrographic seizures should be promptly treated, as ongoing epileptiform activity may aggravate secondary neuronal injury. Continuous EEG monitoring is particularly valuable in comatose patients [48].

#### 3.5.4. Ethical Considerations in Neurological Uncertainty

Neurologic uncertainty is intrinsic to HCA and mandates caution in early prognostication. Premature withdrawal of life-sustaining therapy should be avoided, and decisions should rely on sustained multimodal evidence obtained after confounding factors have resolved [56].

### 3.6. Complications of Extracorporeal Rewarming

Extracorporeal life support in HCA is associated with the full spectrum of complications observed in V-A ECMO, yet several pathophysiological features of profound hypothermia may modify both risk profile and clinical presentation.

#### 3.6.1. Hemorrhagic and Coagulation Disorders

Severe hypothermia profoundly alters hemostasis through platelet dysfunction, impaired coagulation enzyme activity and fibrinolytic imbalance. Upon rewarming, the coexistence of hypothermia-induced coagulopathy, hemodilution and systemic anticoagulation required for ECMO may significantly increase bleeding risk. Cannulation-site hemorrhage, retroperitoneal bleeding, gastrointestinal bleeding and intracranial hemorrhage represent major concerns [14,57,58,59,60]. In addition, rapid thermal shifts may exacerbate endothelial activation and capillary leak, contributing to diffuse oozing [14]. Careful monitoring of coagulation parameters, individualized anticoagulation strategies and frequent reassessment of bleeding risk are therefore essential during the rewarming phase.

#### 3.6.2. Thrombotic Events

Paradoxically, hypothermia is also associated with increased blood viscosity and a prothrombotic tendency. Sluggish microcirculatory flow during low-flow states, combined with circuit-related thrombogenicity, may predispose to thrombus formation within the extracorporeal circuit or native vasculature [61,62]. Balancing hemorrhagic and thrombotic risk in this population requires dynamic adjustment of anticoagulation intensity, particularly during the transition from profound hypothermia to normothermia when coagulation kinetics change rapidly [63,64,65].

#### 3.6.3. Limb Ischemia and Vascular Complications

Peripheral femoro-femoral cannulation carries a risk of distal limb ischemia, which may be exacerbated by cold-induced vasoconstriction and microvascular dysfunction. Routine placement of a distal perfusion cannula and close clinical surveillance of limb perfusion are strongly recommended. Vascular injury during cannulation may be more frequent in hypothermic patients due to increased arterial stiffness and fragile vascular walls. Ultrasound-guided percutaneous access is therefore preferable whenever feasible [6,66,67,68].

#### 3.6.4. Multiorgan and Neurological Complications During Extracorporeal Support

Extracorporeal rewarming may be complicated by clinically significant organ dysfunction requiring prolonged intensive care support. Acute kidney injury is common following prolonged low-flow states and may necessitate renal replacement therapy. Acute lung injury, particularly in patients with aspiration or avalanche-related hypoxia, may complicate ventilatory management despite restoration of systemic perfusion. Persistent myocardial stunning can prolong the need for circulatory support even after normothermia is achieved. In addition to primary hypoxic–ischemic injury, extracorporeal support introduces specific neurological risks. Anticoagulation exposure, hypothermia-related coagulopathy and rapid hemodynamic shifts during rewarming increase the risk of intracranial hemorrhage and secondary cerebral injury. Fluctuations in perfusion pressure and oxygenation may further contribute to cerebral vulnerability in the early post-rewarming phase. Early recognition of evolving organ dysfunction, careful anticoagulation management and structured neurological surveillance are therefore integral to post-ECMO care in HCA [69,70,71,72,73].

### 3.7. Ethical Boundaries and Futility

Severe HCA challenges traditional concepts of medical futility. The well-known principle that “no one is dead until warm and dead” reflects the unique reversibility associated with profound hypothermia and has justifiably shaped modern resuscitation strategies [74,75]. However, this principle should not be interpreted as an unconditional mandate for unlimited extracorporeal support. V-A ECMO in hypothermic cardiac arrest represents a resource-intensive intervention requiring specialized personnel, infrastructure and coordination. While prolonged resuscitation may result in meaningful neurologic recovery, extracorporeal support must remain anchored to physiological plausibility, proportionality of care and system responsibility [75,76,77].

#### 3.7.1. Physiological Plausibility and Limits of Reversibility

Hypothermia provides metabolic protection, but it does not confer immunity from irreversible injury. Decisions regarding continuation or discontinuation of extracorporeal life support should integrate: response to controlled rewarming (including absence of ROSC at 32–35 °C); evidence of catastrophic hemorrhage or non-survivable injury; and persistent refractory multiorgan failure despite optimized support. Futility in this context should not be defined by time alone, but by the convergence of physiological non-recovery and absence of reversibility [6,40].

#### 3.7.2. Prognostic Uncertainty and Temporal Prudence

One of the defining features of hypothermic cardiac arrest is prognostic uncertainty. Delayed drug metabolism, temperature-dependent electrophysiology and reperfusion dynamics complicate early assessment. For this reason, temporal prudence is essential: decisions to withdraw support should rarely be made during the immediate post-rewarming phase unless clear non-survivable conditions are present. Structured reassessment at predefined intervals, incorporating neurologic evolution, hemodynamic trajectory and organ function provides a more ethically sound framework than isolated early determinations [4,5,40,75,78].

#### 3.7.3. Resource Allocation and System Responsibility

Extracorporeal resuscitation requires substantial institutional resources. In regionalized ECMO systems, patient selection inevitably intersects with considerations of capacity and competing clinical demands. Transparent institutional protocols, shared decision-making pathways and predefined escalation and de-escalation criteria may reduce variability and support equitable access to care. Balancing individual rescue potential with responsible resource stewardship is particularly relevant in settings with limited ECMO availability. Ethical frameworks should therefore incorporate both patient-centered benefit and system-level sustainability [79,80,81,82].

#### 3.7.4. Communication and Shared Decision-Making

Given the inherent uncertainty in HCA, clear communication with families is essential. Clinicians should explain the potential for delayed recovery, the rationale of ongoing support and the criteria that would prompt reassessment. Shared decision-making grounded in honesty about uncertainty fosters trust and aligns treatment goals with patient values [75].

## 4. Discussion

HCA represents a unique paradigm in resuscitation medicine in which profound physiological collapse coexists with the potential for complete neurological recovery. Unlike normothermic cardiac arrest, severe accidental hypothermia profoundly suppresses cellular metabolism and oxygen consumption, thereby extending the temporal window of reversible circulatory arrest [1,18,34]. The introduction of extracorporeal life support has fundamentally transformed the management of these patients, enabling controlled restoration of systemic perfusion and oxygen delivery even after prolonged periods of apparent clinical death [83]. Consequently, the management of HCA should not be interpreted as a process of temperature correction, but rather as a complex physiological transition from hypometabolic arrest to reperfusion [1,18]. From this perspective, extracorporeal rewarming may be more accurately conceptualized as a controlled reperfusion of a metabolically suppressed organism. Despite increasing recognition of extracorporeal rewarming as the preferred treatment for severe HCA, its application in everyday clinical practice remains heterogenous across countries and healthcare systems. High-volume ECMO centers, particularly in regions with well-established hypothermia rescue systems such as central Europe and Scandinavian countries, have developed structured referral pathways and prehospital triage strategies that facilitate timely access to extracorporeal rewarming [1,3,5,15,84]. In contrast, in many healthcare settings, limited availability of ECMO resources, logistical constraints and lack of standardized protocols may restrict its implementation. As a result, the likelihood of receiving extracorporeal rewarming is often determined not only by patient-related factors, but also by system-level organization and access to specialized care. These observations underscore that the effectiveness of extracorporeal rewarming extends beyond physiological suitability and depends critically on healthcare system readiness. Increasing awareness, development of regional ECMO networks and implementation of structured clinical pathways may facilitate broader and more appropriate use of this life-saving strategy, ultimately improving survival and neurological outcomes.

The restoration of circulation in profoundly hypothermic patients is accompanied by a cascade of pathophysiological processes, including myocardial stunning, systemic vasoplegia, microcirculatory dysfunction and oxidative stress [1,34,85]. V-A ECMO provides a unique platform to modulate these processes through controlled perfusion flows, adjustable oxygen delivery and gradual thermal normalization [86,87]. This capacity to regulate systemic perfusion distinguishes extracorporeal rewarming from conventional resuscitation strategies and may partly explain the remarkable neurological outcomes observed in selected patients with severe accidental hypothermia [88,89]. This physiological rationale is also reflected in clinical outcomes. Survival with favorable neurological status after HCA treated with extracorporeal rewarming has been reported in approximately 40–50% of carefully selected patients, a rate that compares favorably with outcomes reported in many cohorts of normothermic cardiac arrest with prolonged resuscitation times. These observations support the concept that profound hypothermia may extend the window of reversible ischemia and justify the aggressive resuscitation strategies recommended in current guidelines [40,89,90]. Current international recommendations have improved the identification of patients who may benefit from extracorporeal rewarming, particularly through the adoption of prognostic tools such as the HOPE score and through structured algorithms [5,6,40]. However, several aspects of ECMO management in hypothermic cardiac arrest remain insufficiently standardized. In particular, the optimal hemodynamic targets during rewarming, strategies to mitigate myocardial dysfunction and vasoplegia, the management of oxygen delivery during reperfusion and the role of adjunctive mechanical unloading strategies remain incompletely defined. Rather than providing definitive answers, this narrative review aims to critically synthesize the available evidence and integrate it with pathophysiological reasoning, highlighting areas of uncertainty and potential direction for future investigations.

The cardiovascular response to rewarming represents one of the most challenging phases of management. Experimental and clinical observations consistently demonstrate that myocardial contractility may remain severely depressed during early reperfusion, even after restoration of systemic circulation. This phenomenon, often referred to as rewarming shock, is characterized by transient myocardial dysfunction, reduced systemic vascular resistance and impaired microcirculatory regulation [91,92]. ECMO support provides temporary circulatory stabilization during this vulnerable phase, allowing progressive recovery of cardiac function while maintaining systemic perfusion [76]. Nevertheless, the optimal strategies to balance ECMO flow, afterload and myocardial workload remain uncertain and require future investigation. Neurological prognostication represents another critical area of uncertainty. The profound metabolic suppression induced by hypothermia alters many of the physiological parameters traditionally used to predict neurological outcome after cardiac arrest. Pharmacokinetics of sedative agents are markedly prolonged, electrophysiological patterns may be difficult to interpret and delayed neurological recovery is well-documented in hypothermic patients. Consequently, prognostic frameworks derived from normothermic cardiac arrest may not be directly applicable in this context [6,18,41,43]. Premature withdrawal of life-sustaining therapy therefore represents a significant ethical and clinical risk, underscoring the need for cautious and delayed prognostic evaluation in patients undergoing extracorporeal rewarming [6,18]. Beyond individual physiological considerations, system-level factors may also significantly influence outcomes in HCA. The availability of regional ECMO centers, structured referral networks and coordinated prehospital triage pathways may determine whether eligible patients reach specialized centers capable of providing extracorporeal resuscitation [93]. In this regard, the management of severe accidental hypothermia should be increasingly viewed within the framework of organized extracorporeal resuscitation systems, similar to those developed for refractory cardiac arrest.

Despite the growing clinical experience with extracorporeal rewarming, the evidence base supporting many aspects of management remains limited. Most available data derive from observational registries, retrospective analyses and heterogeneous case series [4,40,87,94,95]. Prospective trials are unlikely to be feasible given the rarity and logistical complexity of severe HCA. Consequently, future progress will likely depend on high-quality prospective international registries, collaborative multicenter studies and improved physiological characterization of reperfusion injury during extracorporeal rewarming. Taken together, the available evidence suggests that HCA should be approached through an integrated framework combining careful patient selection, physiology-guided reperfusion and cautious neurological assessment. Extracorporeal rewarming does not simply restore body temperature but orchestrates a controlled transition from metabolic arrest to systemic reperfusion. Understanding and optimizing this transition may represent the key to further improving outcomes in one of the most extraordinary scenarios in modern resuscitation medicine.

## 5. Conclusions

HCA challenges conventional paradigms of cardiac arrest management by combining extreme physiological derangement with potential reversibility. V-A ECMO has become central to contemporary treatment; however, optimal outcomes require more than timely initiation. A physiology-guided, system-aware and ethically grounded approach, integrating dynamic patient selection, controlled reperfusion and cautious neurologic assessment, may better translate the biological reversibility of hypothermia into survival with preserved neurological function.

## 6. Future Directions

Future research should focus on refining dynamic risk models, exploring metabolic and microcirculatory monitoring during rewarming and developing pragmatic multicenter registries capable of capturing both physiological and system-level variables. Standardization of data collection across regional ECMO networks may facilitate recalibration of existing prognostic models and identification of patient subgroups most likely to benefit from extracorporeal support. Advances in personalized perfusion strategies and integration of real-time physiologic monitoring technologies may further enhance the safety and effectiveness of extracorporeal resuscitation in this unique population.

## Figures and Tables

**Table 1:** Physiology-oriented decision framework for ECMO consideration in hypothermic cardiac arrest. ECMO: extracorporeal membrane oxygenation; CPR: cardiopulmonary resuscitation; HOPE: hypothermia outcome prediction after ECLS; VF: ventricular fibrillation, VT: venticular tachycardia; PEA: pulseless electrical activity.

Domain	Green Zone (ECMO Strongly Considered)	Gray Zone (Individualized Decision)	Red Zone (ECMO Generally Not Recommended)	Clinical Interpretation
Core temperature	≤30 °C	30–32 °C	>32 °C with clear normothermic etiology of arrest	Profound hypothermia supports the hypothesis of temperature-mediated metabolic suppression
Mechanism of hypothermia	Primary environmental exposure (avalanche, cold-water immersion, outdoor exposure)	Unclear chronology between arrest and cooling	Cardiac arrest clearly preceding cooling	Arrest caused by hypothermia is more likely reversible
Serum potassium	<8 mmol/L	8–12 mmol/L	>12 mmol/L (confirmed, non-hemolyzed sample)	Marker of cellular lysis and prolonged hypoxia; must be interpreted in clinical context
Trauma	No major trauma	Suspected non-lethal trauma	Massive trauma incompatible with survival	Trauma-related arrest reflects different pathophysiology
No-flow time	Witnessed collapse or short no-flow interval	Unwitnessed arrest with plausible rapid cooling	Prolonged no-flow in normothermic conditions	Hypothermia may modify the time–injury relationship
Low-flow quality (CPR)	Continuous high-quality CPR (mechanical CPR preferred)	Intermittent CPR in hostile environment	Prolonged ineffective CPR	Quality of perfusion may be more relevant than duration alone
Initial rhythm	VF/VT, PEA	Asystole	Asystole alone is not exclusion	Initial rhythm has limited prognostic discrimination in hypothermia
Avalanche burial	Airway patent	Unknown	Burial > 60 min with airway obstruction	Airway patency and burial duration help distinguish primary hypoxic arrest from hypothermia-mediated arrest
HOPE score	≥10% predicted survival	5–10%	<5%	Prognostic support tool that should not replace clinical judgment
System feasibility	ECMO center available within reasonable transport time	Delayed transport but sustained CPR feasible	No ECMO access or unsafe cannulation conditions	Logistics and system organization strongly influence survival

This framework represents conceptual synthesis of physiological and clinical factors commonly considered in hypothermic cardiac arrest. It is not intended as a validated prognostic algorithm but as a practical support for structured clinical decision-making and should be considered hypothesis-generating, requiring prospective validation.

**Table 2:** Physiology-guided management targets during extracorporeal rewarming. NIRS: near-infrared spectroscopy.

Parameter	Suggested Target	Rationale
Mean arterial pressure	50–70 mmHg	Maintain cerebral perfusion while avoiding excessive afterload
ECMO flow	Adequate to ensure systemic perfusion (usually 3–4 L/min)	Supports oxygen delivery during myocardial recovery
Rewarming rate	Up to 5 °C/h until ≃30 °C, then individualized	Avoids abrupt metabolic and vascular shifts
Circuit thermal gradient	≤4 °C (venous inflow vs. arterial outlet)	Reduces hemolysis and neurologic risks
Oxygenation	Avoid hypoxia and hyperoxia	Limits oxidative stress during reperfusion
Acid-base strategy	Alpha-stat management	Preserves cerebral autoregulation
Cerebral monitoring	Right radial arterial sampling ± NIRS	Detects differential hypoxia

## Data Availability

No new data were created or analyzed in this study.

## Abbreviations

The following abbreviations are used in this manuscript:

HCA	Hypothermic cardiac arrest
V-A ECMO	Veno-arterial extracorporeal membrane oxygenation
CPR	Cardiopulmonary resuscitation
ECPR	Extracorporeal cardiopulmonary resuscitation
EEG	Electroencephalography
F-F	Femoro-femoral
